# Optimizing Potassium-Based Activator Formulation for Balanced Reactivity, Flowability, Setting Time and Mechanical Performance of Alkali-Activated Materials

**DOI:** 10.3390/ma19122604

**Published:** 2026-06-17

**Authors:** Gulsen Nazerian, Jun Gu, Tine Tysmans, Hubert Rahier

**Affiliations:** 1 Sustainable Materials Engineering (SUME) Research Group, Department of Materials and Chemistry (MACH), Vrije Universiteit Brussel (VUB), Pleinlaan 2, 1050 Brussels, Belgium; jun.gu@vub.be (J.G.); hubert.rahier@vub.be (H.R.); 2 Research Group Mechanics of Advanced Materials and Structures (M^2^S), Department Mechanics of Materials and Constructions (MeMC), Vrije Universiteit Brussel (VUB), Pleinlaan 2, 1050 Brussels, Belgium; tine.tysmans@vub.be

**Keywords:** alkali-activated materials (AAM), ground granulated blast furnace slag (GGBFS), potassium silicate modulus, reactivity, setting time, flowability, mechanical properties

## Abstract

Alkali-activated materials (AAMs) based on industrial by-products, such as ground granulated blast furnace slag (GGBFS), are increasingly considered sustainable alternatives to Ordinary Portland Cement (OPC) due to their lower environmental impact and favorable mechanical performance. Among the key parameters controlling the behavior of alkali-activated systems, the chemical composition and modulus of the alkaline activator play critical roles in determining the reaction kinetics and material properties. This study investigates the influence of potassium silicate modulus (Ms), defined as the molar ratio of silica to alkali oxide (SiO_2_/K_2_O), on the reactivity, setting time, flowability, and mechanical properties of alkali-activated slag pastes. Potassium silicate solutions with moduli ranging from 1.0 to 2.5 were used as activators for GGBFS. Paste specimens with different activator moduli were prepared and cured at 20 °C and 75% relative humidity for mechanical testing. The results show that the activator modulus significantly affects the fresh properties, particularly at higher modulus values. Increasing the modulus delays reactivity and prolongs the setting time, whereas the flowability of the fresh paste decreases. Nevertheless, the flowability of the mixtures remained sufficient to allow proper penetration between open textile meshes, which is essential for textile-reinforced cement/concrete (TRC) applications. No clear systematic trends were observed in the mechanical properties, including the elastic modulus, flexural strength, and compressive strength.

## 1. Introduction

Alkali-activated slag (AAS) is increasingly recognized as a sustainable alternative to ordinary Portland cement (OPC), offering favorable properties such as rapid strength development and high durability while reducing environmental impact by reusing industrial by-products such as ground granulated blast furnace slag (GGBFS) [1]. The choice of alkaline activator critically influences the setting behavior, microstructure, mechanical properties, and durability of AAS. Common activators include sodium hydroxide (NaOH), sodium carbonate (Na_2_CO_3_), sodium silicate (water glass), potassium hydroxide (KOH), and potassium silicate. Each has distinct effects on the reaction kinetics and final performance of the alkali-activated slags. For example, NaOH-activated slags typically form more polymerized calcium silicate hydrate networks and show relatively faster setting compared with sodium carbonate activation, where the initial formation of sodium calcium carbonate retards the reaction and extends the setting time [2]. Sodium carbonate activators offer economic and environmental benefits; however, their main drawback is poor early-age strength unless supplemented with additives such as calcium oxide or sodium silicate, which enhance the early strength development and reaction kinetics [3]. The blend of sodium carbonate and sodium silicate efficiently activates slag, improving early strength development by increasing alkalinity and providing extra silicate for reaction [4].

In potassium-based systems, KOH is often combined with potassium silicate to adjust alkalinity. The addition of KOH increases pH and promotes further dissolution of silicate species, ensuring sufficient alkalinity for effective slag dissolution and network formation. This balance between alkalinity and available silicate species is a key factor governing reaction kinetics and binder formation in alkali-activated systems. A number of studies have highlighted systematic differences between sodium- and potassium-based activation routes. Hosan et al. [5] demonstrated that fly ash geopolymers activated with potassium-based solutions exhibit greater thermal stability than sodium-based counterparts, showing higher residual compressive strength, reduced mass loss, lower shrinkage, and minimal cracking. Bakthavatchalam et al. [6] reported that sodium silicate solutions have higher viscosity than potassium silicate solutions, which leads to lower strength and higher porosity. Zhao et al. [7] demonstrated that potassium silicate solution is a promising alternative to sodium silicate for activating alkali-activated materials (AAMs), avoiding issues such as efflorescence and excessive expansion. Similar findings were reported by Maghsooldoorad et al. [8], who observed that potassium-based activators, including KOH and potassium silicate, resulted in reduced efflorescence compared to sodium-based activators in alkali-activated phosphorus slag cement. This reduction in efflorescence suggests enhanced durability, particularly regarding leaching resistance and surface deposition phenomena. Despite these advantages, potassium-based activators are more expensive than sodium-based ones [9], and further research is needed to explain the synergistic effect of K_2_O content and the activator modulus (Ms), which is described as the molar ratio of silica to alkali oxide (SiO_2_/K_2_O), on the hydration kinetics, phase evolution, and long-term performance of potassium-activated slag binders. A comprehensive understanding of the synergistic effect of K_2_O content and Ms on AAS paste properties is still lacking. Although alkali-activated slag systems have been widely studied, much of the literature focuses on blended precursor systems incorporating fly ash, metakaolin, or other supplementary materials. In contrast, investigations centered specifically on blast furnace slag activated predominantly with potassium silicate remain comparatively limited. Recent work by Chen et al. (2025) [10] explored potassium silicate-activated slag as a cement-free binder for 3D concrete printing. They proposed a mix design strategy addressing rapid setting and poor workability, demonstrating that properly optimized formulations can achieve both printability and mechanical performance. This highlights the importance of balancing fresh-state rheology with mechanical performance in potassium-activated systems.

Markusík et al. [11] conducted a detailed investigation of the rheological behavior of alkali-activated slag systems, focusing on the influence of activator type and molarity through oscillatory amplitude testing. Their results indicated that silicate-based activators generally enhance flow behavior compared with hydroxide-based alternatives, with potassium silicate showing especially advantageous effects. Their results further indicated an optimal activator concentration range, beyond which rheological performance deteriorates, emphasizing the sensitivity of fresh-state properties to activator dosage.

Several studies have also compared potassium- and sodium-based activation routes. Omur et al. [12] examined BFS mortars activated using either KOH or NaOH and reported that KOH activation resulted in improved workability and higher compressive strength. In contrast, NaOH activation led to shorter setting times, reduced water absorption, and lower drying shrinkage. The authors further observed that increasing the activator molarity reduced both yield stress and plastic viscosity, underscoring the strong influence of alkali chemistry on the rheological properties of fresh mixtures.

In a related study, Singh et al. [13] evaluated mechanochemically treated low-alkali activated slag concretes using potassium- and sodium-based activators. Their findings indicated that potassium activation promoted the formation of a denser microstructure, which translated into enhanced long-term mechanical performance and durability, including reduced permeability and improved resistance to chemically aggressive environments. These results suggest that potassium-based activators may provide performance and sustainability benefits, particularly when combined with mechanochemical treatment.

Although not limited solely to potassium silicate systems, Panda et al. [14] showed that incorporating potassium silicate into blended alkaline activator formulations significantly enhanced the workability, strength development, and durability of GGBFS-based self-compacting geopolymer concrete. Similarly, Agista et al. (2024) [15] reported that slag-based geopolymer systems activated with potassium silicate exhibited favorable rheological and mechanical behavior for low-temperature well-cementing applications, provided that appropriate precursor selection and particle size distribution were ensured.

Surehail and his colleagues [16] showed that the nature of the cation and the physical form of the activator in K–Si-activated systems play a key role in controlling the dissolution kinetics and the transport of ionic species within the system. This leads to notable variations in early-age reaction pathways. Overall, the study highlights that while potassium activators enhance workability and early reaction kinetics, sodium activators are more effective in achieving superior long-term mechanical performance.

Good flowability is required if the paste or mortar has to flow between an open textile with a typical mesh width of approximately 1 cm. As potassium silicate solutions have lower viscosity than sodium silicates, they are preferred for producing (textile-reinforced concrete) TRC. Although potassium silicate is about 30% more expensive than sodium silicate on a unit mass basis, and even more on the molar mass basis [17], its impact on the overall cost of the TRC formulation is limited because the activator represents only a relatively small fraction of the total solids (activator-to-solid ratio = 0.27). As a result, the increase in total material cost remains modest. From a cost–performance perspective, this additional cost is compensated by practical advantages relevant to TRC applications. Concrete slabs can be made thinner because no extra concrete cover is needed to protect the steel reinforcement from corrosion. Potassium silicate improves fresh-state workability and reduces efflorescence, which are critical for achieving uniform impregnation of textile reinforcement, maintaining good matrix continuity, esthetics, and durability. These aspects directly affect the quality and reliability of the composite in practice.

In addition, the mechanical performance, particularly the compressive strength, was evaluated in the hardened state. Different activator moduli (1–2.5) were examined to determine their effects on key material properties. The binder system was first developed at the paste level as a preliminary step toward producing mortars suitable for textile-reinforced concrete TRC applications. Studying the material at this scale allows fundamental behavior of the system to be evaluated without the influence of aggregates, enabling a clearer assessment of reactivity, setting time, rheological behavior, and mechanical properties. This approach provides a reliable basis for the subsequent development and optimization of mortar formulations.

Therefore, the novelty of our work lies in providing a systematic optimization of potassium-based activator formulations for alkali-activated GGBFS, focusing simultaneously on reactivity, flowability, setting time, and mechanical performance. While potassium activators have been mentioned in previous studies, existing literature typically examines only isolated properties or focuses primarily on sodium-based systems. In contrast, this study integrates fresh- and hardened-state performance within a unified optimization framework.

This study aimed to investigate the influence of the modulus of the potassium silicate activator on the behavior of alkali-activated slag systems and to identify a suitable paste composition for use as a binder phase in TRC mortars. The work focuses exclusively on paste-level behavior, providing foundational knowledge for future mortar and TRC-related studies. Particular attention is given to fresh-state characteristics, including setting time and workability, due to their importance in handling and practical applications.

## 2. Materials and Methods

### 2.1. Materials

GGBFS used as the main precursor was provided by Ecocem fromEcocem Benelux B.V., Moerdijk, The Netherlands. Powder samples were collected from multiple bags to ensure representative sampling, as the reactivity varied between bags. The powders were then combined and homogenized thoroughly to reduce variability across sources. This homogenized mixture was used in all subsequent experiments. The chemical composition obtained from X-ray fluorescence (S4 PIONEER XRF spectrometer (Bruker AXS GmbH, Karlsruhe, Germany)) is presented in Table 1. The analysis was performed solely on powdered samples that were dried to a constant mass and then pressed into pellets for measurement using internal calibration in the standardless mode. Physical properties of GGBFS are summarized in Table 2.

**Figure 1:**
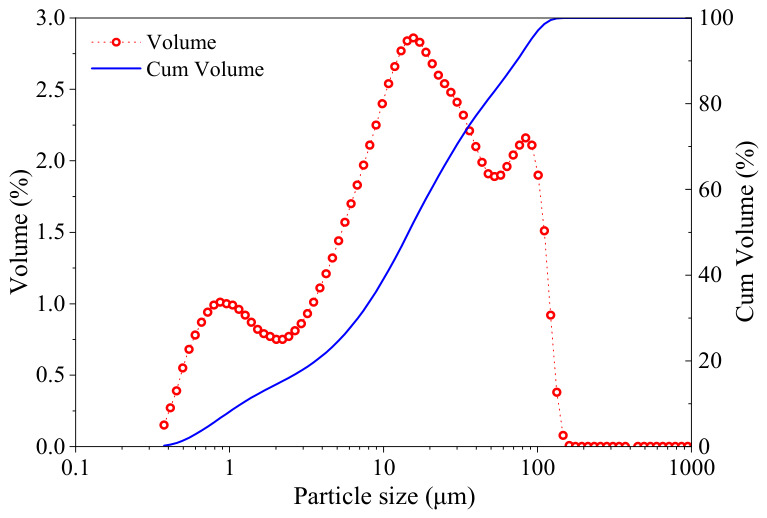
Particle size distribution curves of GGBFS.

**Figure 2:**
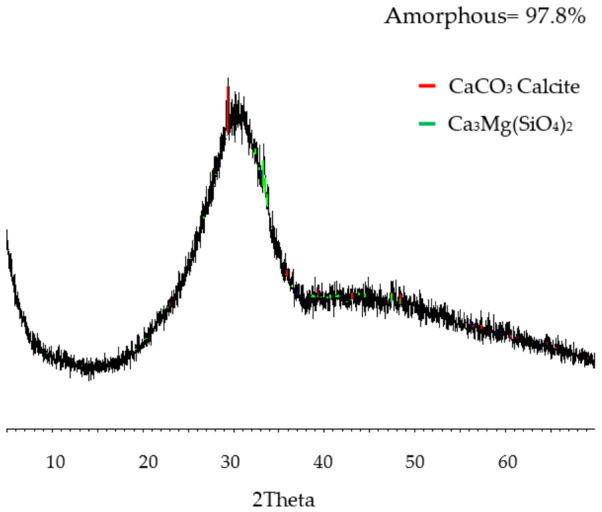
X-ray Diffraction analysis of GGBFS showing that it is almost purely amorphous.

The particle size distribution of GGBFS, determined using a laser particle size analyzer (Beckman Coulter LS 13 320, Brea, CA, USA), is shown in Figure 1. The medium particle size (d50) was 9.9 µm. The XRD diffractogram (D8 Advance Eco, Bruker Corporation, Billerica, MA, USA) shows that GGBFS is 97.8% amorphous (Figure 2) with a few crystalline features ascribed to Ca(CO_3_) Calcite and Ca_3_Mg (SiO_4_)_2_ merwinite.

For the activation of GGBFS, potassium hydroxide (KOH) with a purity of 85% (Sigma Aldrich, St. Louis, MO, USA) and potassium silicate solution (SilmacoNV, Lanaken, Belgium) were used. The chemical composition of the potassium silicate solution was K_2_O = 15.0 wt.%, SiO_2_ = 27.2 wt.%, and H_2_O = 57.7 wt.%.

### 2.2. Mix Design

KOH pellets were weighed, dissolved in potassium silicate, and stirred until completely dissolved. A fresh solution was prepared 24 h prior to the synthesis of the AAMs, and left at ambient temperature to cool. To evaluate the influence of the activator modulus (Ms) on paste behavior, a preliminary investigation was conducted over a broad modulus range from 1.0 to 2.5, with increments of 0.25 (1.00, 1.25, 1.50, 1.75, 2.00, 2.25, and 2.50). Based on the preliminary results and observations, the effective modulus range was narrowed down to Ms = 2.00–2.35 (with an interval of 0.05). To understand the effect of different silicate moduli on GGBFS-based alkali-activated materials, eight groups of samples were prepared (Table 3).

### 2.3. Mixing, Casting, and Curing

The prepared solution was added to the GGBFS and mixed with a high-speed mixer for approximately 2 min to obtain a homogenous mixture. The paste was then poured into the molds with a size of 40 × 40 × 160 mm^3^, vibrated to remove bubbles, and covered with plastic to prevent water evaporation. After 24 h, the samples were demolded, wrapped in plastic films, and stored at 20 °C and 75% humidity until the designated compressive and flexural strength tests. After 28 days, the specimens exhibited a mass loss of approximately 0.2% due to moisture loss. All these steps were repeated to prepare pastes with different solution moduli. The pH of the activators was measured using a pH meter (Mettler Toledo, Greifensee, Switzerland). Prior to this, multiple experiments were conducted to determine the optimum solution-to-binder ratio. The GGBFS was mixed with two different solutions (Ms 2.15 and 2.25) at various ratios (with repetition of 2), ranging from 0.3 to 1.0, and the reactivity and flowability of the mixtures were observed. Isothermal calorimetry results indicated that a liquid-to-solid ratio of 0.8 yielded the highest heat release while maintaining adequate flowability for the intended application.

### 2.4. Experimental Methods

#### 2.4.1. Slump Flow Test

The flowability of the fresh paste was investigated using a conical cone with a height of 60 ± 0.5 mm, top diameter of 70 ± 0.5 mm, and bottom diameter of 100 ± 0.5 mm, as described in ASTM C230 [18]. Prior to each test, the paste was mixed for 2 min and then gradually poured into the cone, which was placed in the center of a clean and lubricated plate. After filling, the cone was slowly lifted to allow the paste to spread. The flow diameter was measured in three perpendicular directions and recorded as the flow spread.

#### 2.4.2. Rheology of Paste

The rheological characterization of the paste was conducted using a core rheometer (TA instruments, New Castle, DE, USA) with a parallel-plate geometry and a plate diameter of 40 mm. The paste was mixed for 2 min and subsequently transferred to the rheometer plate. The measurement gap (500 µm) was then set, and prior to measurement, a resting (delay) time of 1 min was applied to allow structural stabilization of the paste before loading. Following this equilibration period, the test was performed at a constant shear rate of 100 s^−1^ for 10 min at 20 °C. This procedure was consistently applied to all paste formulations, including those with different moduli, to ensure the comparability of the rheological response across the mixtures.

#### 2.4.3. Setting Time

##### Ultrasonic Pulse Velocity (UPV)

A non-destructive ultrasonic pulse transmission technique was used to evaluate the setting and hardening processes of cement-based materials. P-wave ultrasonic transmission measurements, according to EN 12504-4 [19], were conducted using a commercially available IP-8 ultrasonic device (UltraTest GmbH, Achim, Germany). The system includes a controller unit connected to a PC capable of supporting up to eight measurement cells. Each cell comprised a high-damping silicone mold, an ultrasonic transmitter-receiver pair, and a temperature sensor. To minimize interference from wave propagation through the mold rather than through the sample, four integrated acoustic dampers were incorporated into the silicone mold. Freshly mixed samples were placed into the mold, kept at a consistent temperature of 20 ± 2 °C, and sealed to prevent moisture loss. The pulse propagation time (t) across the distance between the transmitter and receiver was recorded to calculate the velocity (v = l/t) through the samples, starting from fresh pastes to solidified material. The time between the start of mixing and the start of the UPV experiment was 4 min. This time was added to the experiment. The experiments were stopped when the velocity stabilized shortly after the final setting time elapsed.

##### Vicat Needle Test

The setting behavior of the alkali-activated paste was evaluated using a Vicat needle apparatus in accordance with the EN 196-3 standard [20]. Immediately after mixing, the fresh paste was cast into molds (top inner diameter: 70 ± 0.5 mm; height: 40 ± 0.2 mm; bottom inner diameter: 80 ± 0.5 mm) and positioned on a base plate. All the experiments were performed at 20 ± 2 °C. Each measurement was conducted in triplicate to ensure reproducibility. The setting times were determined automatically using a Vicat device with a data recording capability. The initial setting time was defined as the period from the mixing of GGBFS with the alkaline activator (“zero time”) to the point at which the needle penetration reached 3 mm, corresponding to a penetration depth of 37 mm. The final setting time was recorded when the needle penetration was reduced to less than 0.5 mm into the specimen.

##### Impulse Excitation Technique IET

**Figure 3:**
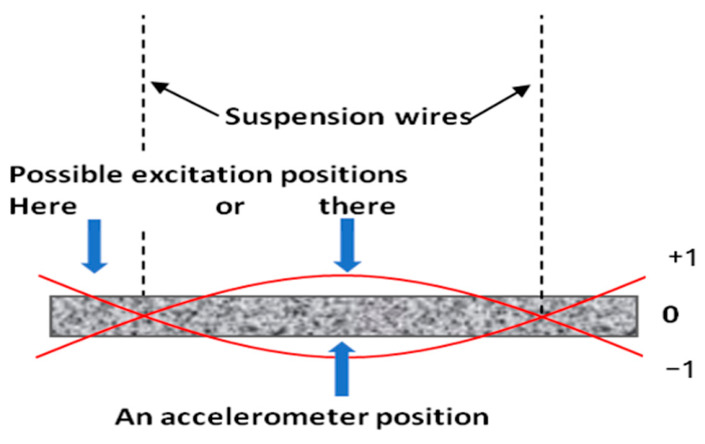
Excitation and measurement position for IET tests on test beams.

The impulse excitation technique (IET) is a non-destructive material characterization technique to determine the elastic properties. It measures the resonant frequencies to calculate the Young’s modulus, shear modulus, Poisson’s ratio and internal friction of predefined shapes, such as rectangular bars and cylindrical rods [21]. The procedure involves striking the specimen with a small hammer and recording the induced vibration using an accelerometer. The resulting vibration signal, known as the impulse response function (IRF), contains information about the resonance frequency and damping ratio. Figure 3 illustrates the excitation of a beam. The beam specimen is suspended at its nodal lines (the set of points with zero vibration amplitudes during a bending modal shape), while the excitation and measurement locations are positioned where the vibration amplitude is sufficiently high, typically near the midpoint of the beam [22].

For a slender beam with a constant rectangular cross-section, Young’s storage modulus E’ (Equation (1)) can be calculated from the measured resonance frequency $f_{r}$ using the analytical expression provided in ASTM E1876-22 [22]:

(1) $$E^{\prime }=0.9465\left(\frac{mf_{r}^{2}}{w}\right){\left(\frac{l}{d}\right)}^{3}K_{IET}$$

Herein, m is the mass of the beam, $f_{r}$ the measured resonance frequency, w is the width, l is the length and d is the thickness. $K_{IET}$ is the correction factor for the finite thickness of the beam, Poisson’s ratio, etc.

**Figure 4:**
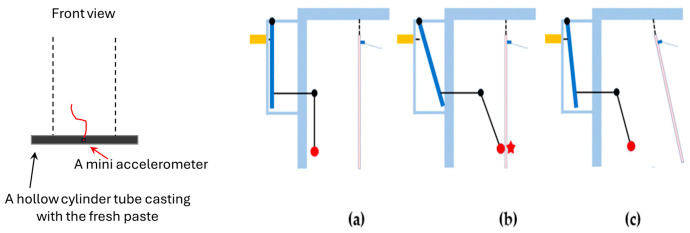
Left: front view of set-up; right: side view of set-up and impulse steps [27]. (**a**) The pendulum is driven by a solenoid, with its main components shown schematically: the lever in dark blue, the excitation beam in black, and the rigid spherical tip in red. (**b**) The pendulum makes contact with the sample (white), and the moment of impact is marked with a red star. (**c**) After striking the sample, the pendulum swings back while the sample undergoes its induced oscillation. The light blue elements correspond to the structural walls of the climate chamber.

To monitor the evolution of the dynamic modulus of the fresh paste during its transition from a fluid state to a hardened material, an extended automated Impulse Excitation Technique (IET) procedure was employed, enabling continuous impulse excitation measurements over 24 h at 2 min intervals inside a climate chamber (Figure 4). For this test, a polycarbonate hollow cylinder was used with an outer diameter of 20 mm, an inner diameter of 16 mm, and a length of 200 mm. Prior to casting the fresh paste, the resonant frequency of the empty hollow cylinder mold was obtained in its fundamental flexural mode, according to ASTM E1876-22. Using the resonant frequency, mass of the mold, and its length, the bending stiffness of the empty cylinder mold was obtained (Equation (2)). The fresh paste was then cast into the hollow cylinder, and both ends were tightly sealed with two silicone stoppers. After casting, a mini-accelerometer was securely attached to the center of the tube mold. The resonant frequencies of the cylinder mold filled with paste were recorded over 24 h, and the total bending stiffness of the filled cylinder mold was subsequently determined. After the 24 h automated IET test, the hardened sample was removed from the cylindrical mold. The mass and geometric dimensions of the demolded sample were then used to convert the stiffness of the paste into its dynamic modulus (Equation (3)), as follows:

EI _empty mold_ ≈ 0.0789 f^2^ L^3^ m (2)

EI _(matrix)_ = EI _(total)_ − EI _(empty mold)_ (3)

where in Equation (2), f is frequency (Hz), L is length (m), and m is mass (kg).

In Equation (3), the area moment of inertia is defined as $I=\frac{\pi r^{4}}{4}$, where r is the diameter of the hardened paste.

##### Determination of Setting Time (UPV and IET)

**Figure 5:**
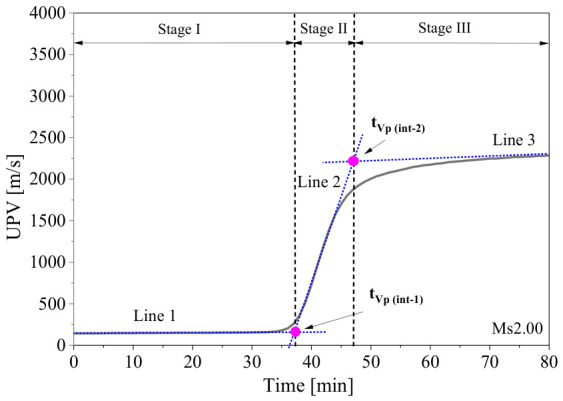
Intersection points on the UPV curve as a function of time for activated paste with Ms 2.00. Line 1 is the extrapolation of the initial plateau region before the velocity rise, line 2 is the tangent drawn through two inflection points of the curve, and line 3 is the extrapolation of the later-stage plateau after the rise.

Figure 5 shows the UPV measurement curve with three distinct stages, as reported in ref. [23]. The first stage, referred to as the dormant period, is characterized by a consistently low ultrasonic pulse velocity, significantly lower than that in air (~340 m/s) and typical alkaline solutions (~1450 m/s). This reduced velocity is primarily attributed to the high concentration of entrapped air bubbles within the fresh paste, which hinders wave transmission through the viscous water-rich suspension [24,25,26].

Following the dormant phase, the ultrasonic pulse velocity rises sharply during the second phase, known as the acceleration stage (Stage II), and then continues to increase more gradually in the third stage, resulting in a characteristic S-shaped curve [28,29]. The setting and hardening of AAMs can be considered a transition from a fluid suspension to a solid network. As this structure forms and becomes increasingly interconnected, the ultrasonic wave propagation improves, resulting in higher velocities.

In Stage III, the rate of velocity increase slows down and begins to plateau. This gradual increase is linked to the continued growth and densification of the network, thus connecting the building blocks to the network through condensation reactions and forming a compact matrix [29]. In this study, the intersection method was used to determine the setting times. The initial setting time is defined as the intersection of lines 1 and 2 (t_Vp(int-1)_). The final setting time corresponds to the intersection of lines 2 and 3 (t_Vp(int-2)_) [30].

The setting time for the IET method was determined using the same method applied to the characteristic increase in the resonant frequency. Specifically, the onset of setting was identified as the point at which the stiffness curve exhibited a sharp and sustained increase, indicating the formation of a continuous solid skeleton.

Various approaches have been proposed to correlate the Vicat initial and final setting times with ultrasonic pulse velocity measurements. One commonly used method involves defining specific threshold values for the ultrasonic velocity to represent the initial and final setting points. However, these threshold values vary considerably across different materials due to differences in composition, testing equipment, and excitation frequencies [29]. An alternative approach, which we applied in this study, focuses on identifying characteristic or inflection points within the ultrasonic velocity curve. The initial setting time is often associated with the point at which the velocity begins to increase, corresponding to the onset of the second stage of the hydration process. The final setting time is linked to either the point of maximum acceleration or the transition into the third stage, where the velocity increase becomes more gradual.

#### 2.4.4. Isothermal Conduction Calorimetry

The heat flow of the paste over time was recorded using a TAM Air isothermal micro-calorimeter (TA Instruments, New Castle, DE, USA) at 20 °C. The heat flow (q) was measured and translated to cumulative heat Q by integration, as follows:

(4) Q(t)=∫q(t).dt

Admix ampoules (20 mL) were used for the isothermal calorimetry measurements. A predetermined mass of GGBFS (4.0 g) and the corresponding mass of activator solution (3.2 g) were introduced into the ampoule. The liquid activator was added using the standard TAM Air injection system, ensuring that the components remained physically separated until the start of the measurement. After thermal equilibration inside the calorimeter, the reaction was initiated by injecting the activator into the powder compartment of the ampoule, allowing in situ mixing and enabling accurate capture of the initial exothermic peak. Sand was used as an inert reference material to compensate for external temperature fluctuations. The heat flow (µW) was recorded for five days and normalized to the total mass of GGBFS.

#### 2.4.5. Compressive and Flexural Strength Testing

The compressive and flexural strengths of the specimens were determined using a compression machine Servo Plus (Matest S.p.A., Bergamo, Italy), with a capacity of 300 kN for compression testing and 15 kN for bending. The flexural strength was measured using a three-point bending test with a span of 100 mm. The compressive and flexural strengths were determined at the age of 28 days. All flexural and compressive strength results are reported as the average values of three and six specimens, respectively. The compressive strength tests were performed on the fractured halves remaining after flexural testing.

## 3. Results and Discussion

After the initial screening, the modulus range was restricted to 2 to 2.35, because mixes with Ms values between 1.0 and 2.0 exhibited excessively rapid setting, which hindered proper casting and handling of the specimens. Conversely, at Ms ≥ 2.40, negligible reactions were observed over several days at room temperature, indicating insufficient activation under these conditions.

### 3.1. Effect of Ms on Heat Evolution Rate

**Figure 6:**
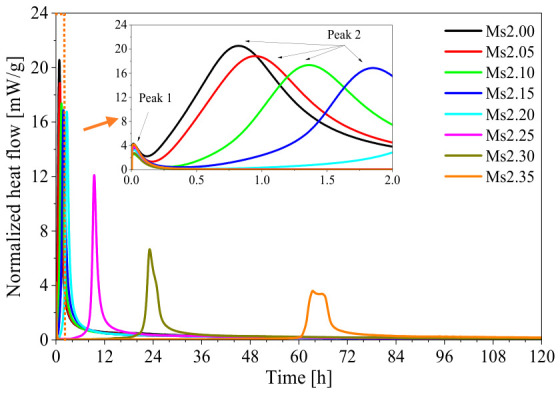
Isothermal heat production curve (20 °C) for activated-GGBFS with different modulus of silicate solution.

Immediately after the activator solution was introduced into the slag powder during isothermal calorimetry, all mixtures exhibited a small but distinct early exothermic peak within the first 20 min (Figure 6). This initial thermal peak is attributed to particle wetting and the rapid dissolution of the outer amorphous layer of GGBFS [16,31]. The heat release at this stage reflects the breakdown of the glassy slag network upon contact with the potassium silicate solution.

A second, more pronounced exothermic peak followed, corresponding to the interactions between the dissolved calcium ions Ca^2+^ and silicate species supplied by the activator. This stage marks the onset of reaction products, primarily the C-(A)-S-H type network, as previously reported [31,32]. The timing and magnitude of this peak varied systematically with the silicate modulus (Ms), indicating a shift in the reaction kinetics as the activator composition changed. Increasing the alkalinity of the activating solution achieved by lowering Ms through the addition of potassium hydroxide significantly enhanced the reactivity of GGBFS. For mixtures with Ms between 2.00 and 2.15, the second peak started relatively early (less than 1 h). As Ms increased beyond 2.20, the second peak progressively shifted to later times, reflecting a slower network formation. In other words, a higher pH, which reflects a higher concentration of free OH^−^ ions (Table 4), accelerates the dissolution of the slag’s amorphous network by breaking Si–O and Al–O bonds, thereby promoting more rapid formation of calcium aluminosilicate hydrate (C-A-S-H) structures, which is consistent with previous reports [16,33,34]. This trend illustrates the significant impact of the modulus on the temporal evolution of hydration processes.

Calorimetric traces further show that systems with lower Ms exhibit markedly sharper and earlier secondary acceleration peaks, indicative of elevated initial dissolution rates and faster overall reaction kinetics compared with systems activated at higher moduli (Ms ≈ 2.25–2.35). Increasing Ms, for example, from 2.20 to 2.30, at a constant water content, reduces the effective alkalinity of the activating solution; this reduction produces broader, temporally delayed autocatalytic exotherms of lower intensity. At the highest modulus examined (Ms = 2.35), a small heat evolution occurred only after 60 h, consistent with a severely retarded reaction. The slow behavior at high Ms values is attributed to the limited availability of free hydroxide ions, which restricts dissolution, thereby retarding further product formation [35]. Overall, a higher activator modulus slowed the rate of reaction product formation and reduced the degree of reaction after the same time, indicating that activator alkalinity is a primary control on the reaction kinetics and the extent of C-A-S-H network development [24,32]. Liu and his team [25] observed the same trend with sodium silicate and sodium hydroxide. In their study, when the SiO_2_ molarity increased, the hydration of the slag slowed down. This retarding effect can be attributed to the decrease in effective OH^−^ availability with increasing SiO_2_ content in the activating solution. Reduced alkalinity retards slag dissolution, resulting in a lower release rate of Ca^2+^ into the pore solution. Consequently, the attainment of Ca^2+^ supersaturation is delayed, which in turn retards the subsequent reaction between Ca^2+^ and silicate species, thereby slowing the formation of the C–A–S–H network.

The mechanistic role of Al in aluminosilicate chain growth was clarified by Salha et al. [36], who demonstrated that Al substitution facilitates oligomerization and promotes the formation of longer aluminosilicate chains under both neutral and anionic conditions. In particular, Al(IV) was identified as the most favorable coordination state for oligomerization, lowering the free-energy barriers associated with trimer and pentamer formation relative to Si-only systems. The study further showed that Al incorporation reduces the energetic cost of chain elongation due to its coordinative flexibility, enabling easier structural rearrangement during Si/Al–O–Si bond formation and subsequent condensation reactions. Under anionic conditions relevant to highly alkaline cementitious environments, Al-containing pathways exhibit lower energetic barriers and a stronger thermodynamic preference for oligomerization than Si-only systems.

**Figure 7:**
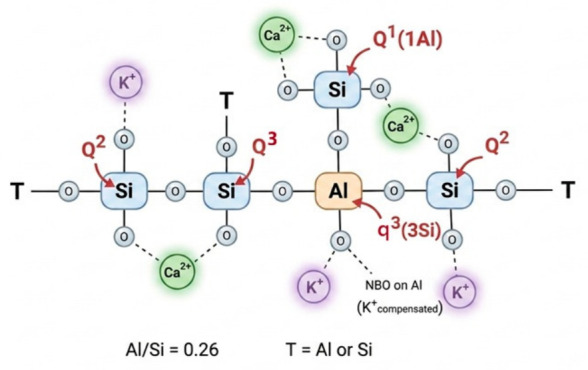
Structural representation of an aluminosilicate material. ‘Q’ represents an SiO_4_ tetrahedron. The subscript denotes the number of links to another network former, while between brackets the number of links to Al is given. ‘q’ represents the same but for AlO_4_ tetrahedra. NBO: Non-Bridging Oxygen.

There is an interesting study [37] that provides insight into the molecular structure of a (C,K)-A-S-H network. However, the study does not cover the compositional range of our study, and the maximum Si/Al ratio is 2, and Na is studied rather than K as a counter ion. Some information from that study can, however, be used, as well as some general knowledge of aluminosilicates. To obtain an idea of what the reaction product would look like on a molecular scale (Figure 7), a few assumptions must be made. It is assumed that all the BFS has reacted, but for the sake of simplicity, only the Al and Si contents are taken into account. It is also assumed that the network is built up homogenously, such that only one structure is formed and no OH groups are drawn. For every 1 Al, there was approximately 4 Si, 1.4 K, and 3.2 Ca (=Ca/Si × 4 Si) in the end product (Mix 1). To calculate the connectivity, the charge introduced by the network modifiers (Ca^2+^ and K^+^) will be calculated per 1 Al and 4 Si present. For charge balance, there is one negative charge per Al if it is assumed that Al is 100% in tetrahedral coordination; this is compensated by K, but there is 0.4 K in excess. Ca gives a total of 6.4 positive charges. Together with the excess K, this gives 6.8 positive charges to be compensated by the four SiO_4_ and one AlO_4_ tetrahedra (in total −20). Thus, there are 6.8 dangling O bonds per 5 (Si, Al) atoms. (Si, Al) thus has an average of 13.8/5 = 2.76 links to other network formers (Q^2.76^ to follow the Engelhardt notation), namely Al or Si. For Al, it is commonly accepted that it is only linked to Si in agreement with the Loewenstein avoidance rule. A representation is given in Figure 7. Since the degree of polymerization is above 2, the final structure is a covalently bonded aluminosilicate network. Considering the many assumptions and the fact that silanol groups will be present, the degree of polymerization will be somewhat lower, and the representation in Figure 7 only provides an impression of the molecular structure.

Atomistic simulations reported in [36] show that tetrahedrally coordinated Al promotes silicate oligomerization by lowering the energetic barriers associated with chain growth. In particular, Al incorporation into the silicate framework facilitates Si–O–Si bond formation through Si–O–Al linkages under both neutral and alkaline conditions, thereby enhancing oligomer growth pathways.

**Figure 8:**
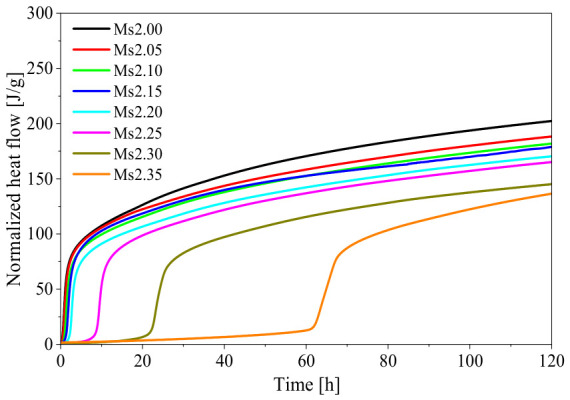
Cumulative heat release for alkali- activated paste with different modulus vs. time.

The mixes activated with a low silicate modulus (Ms: 2.00, 2.05, 2.10, and 2.15) exhibited the fastest early heat evolution, with a short induction period and a steep initial rise in cumulative heat, reflecting rapid slag dissolution and early C-A-S-H formation (Figure 8). After this stage, the heat flow decreased but remained positive, leading to a steady increase in the cumulative heat. This sustained heat release reflects the progressive structural build-up and reorganization of the C-A-S-H network, which continues even after the initial dissolution. The presence of soluble silicate promotes extended condensation reactions and network densification, resulting in a prolonged period of low-intensity but continuous heat evolution. The mixture with the highest amount of potassium hydroxide exhibited the highest total reaction heat for 5 days, reaching approximately 202 J/g. As the modulus increased, the curves became progressively flatter and more delayed; the onset of rapid heat release shifted to later times, and the early-age cumulative heat decreased, indicating that higher silicate content slowed the initial dissolution and reaction kinetics.

### 3.2. Effect of Ms on Setting Time

#### 3.2.1. Temporal Evolution of Ultrasonic Velocity

**Figure 9:**
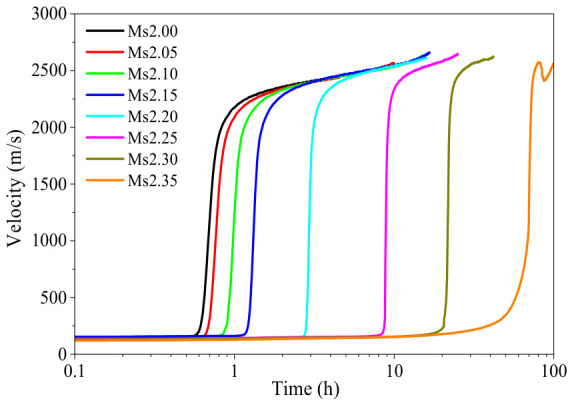
Ultrasonic velocity curve of GGBFS mixtures, time is in logarithmic scale (20 °C).

The initial and final setting times of fresh pastes activated using potassium silicate solutions with different Ms, along with their corresponding UPV, are presented in Figure 9. The setting behavior of the alkali-activated pastes was influenced by the Ms of the activating solution, which is consistent with previous calorimetric observations.

In the initial series of experiments for Ms 2.35, a distinct drop in the measured response was observed, and this behavior was reproduced when the test was repeated under the same conditions. However, when the experiment was repeated several weeks later using the same protocol, this drop was no longer observed. One possible explanation for the observed decrease in elastic modulus is the formation of a defect, such as a microcrack, during hardening. As the reaction proceeds and the microstructure continues to develop, ongoing network formation may compensate for this temporary loss of stiffness. This could explain the subsequent increase in modulus and the apparent recovery from the observed dip.

**Figure 10:**
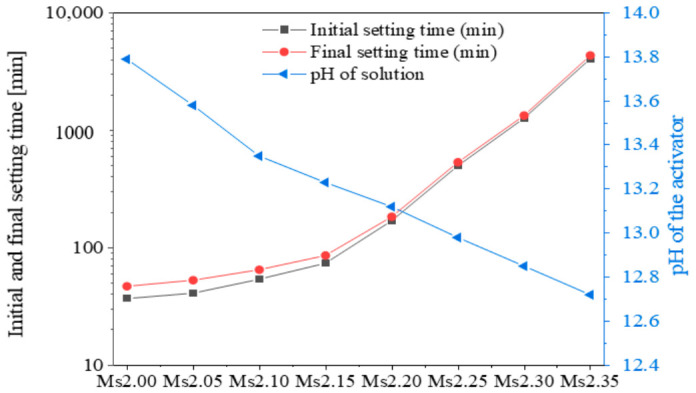
Comparison of initial and final setting time of activated paste (in logarithmic scale) measured by UPV and pH of activator.

The results shown in Figure 10 indicate that a higher silicate modulus corresponds to lower pH levels in the activator solution. A high pH increases the solubility of aluminosilicate anions in the solution while decreasing the Ca^2+^ Concentration. The impact of these chemical processes is further clarified by Daia et al. [38], who noted that while moderate amounts of soluble silicate facilitate the rapid release and reaction of Ca^2+^ ions from GGBFS, excessive silicate levels can reduce the alkalinity and alter the reaction kinetics. Conversely, lower Ms values, achieved through higher potassium hydroxide content, increase the alkalinity of the activating solution, thereby enhancing the dissolution of GGBFS by providing a higher concentration of OH^−^ ions. This leads to a more rapid generation of C-(A)-S-H and shortens the setting time, supporting faster hardening of the paste. Thus, a lower SiO_2_/K_2_O ratio accelerates the reaction and microstructural development, whereas a higher ratio tends to retard these processes [39].

#### 3.2.2. Vicat Setting Time

**Figure 11:**
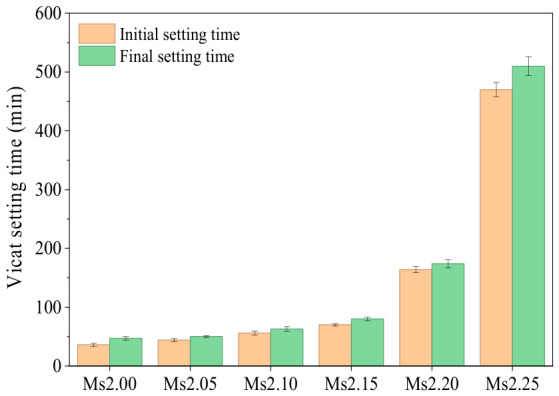
Vicat setting times of alkali-activated pastes.

The influence of SiO_2_ and K_2_O on the setting time was investigated by adjusting their concentrations. Both the initial and final setting times of the mixtures increased with an increase in the modulus of the potassium silicate activator (Figure 11). This behavior can be attributed to the fact that, at a lower modulus, the formation of the initial calcium silicate hydrate (C–S–H) network is accelerated. This acceleration results from the enhanced interaction between the calcium ions released from the GGBFS and silicate species in the pore solution, which promotes the hardening of the alkali-activated paste [40]. The setting times of the mixes with Ms 2.30 and 2.35 were not determined because their setting was excessively delayed (exceeding one day for Ms 2.30 and more than two days for Ms 2.35).

### 3.3. Effect of Ms on Flowability

**Figure 12:**
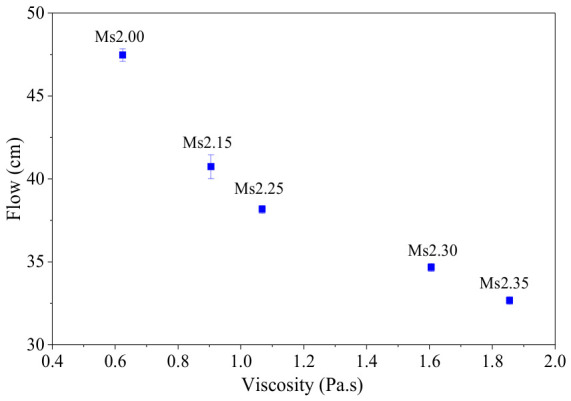
Correlation between Flow and viscosity in different moduli. The error bars for the last three modulus values were small, not sticking out of the symbol.

The correlation between the rheometer results and the flowability of the pastes with different moduli of the activator is shown in Figure 12. The viscosity was measured 6 min after placing the sample on the geometry. This timing is consistent with the flow table procedure, in which approximately 6 min elapse from the initial mixing to the completion of the measurement, including mold removal. The flow values indicated a high level of workability, suggesting that the GGBFS paste possessed sufficient fluidity for effective placement and consolidation in TRC applications. The results showed a reduction in the flowability and an increase in the viscosity of the GGBFS-based paste with increasing activator modulus. This can be attributed to the fact that a higher SiO_2_/K_2_O molar ratio leads to an increased concentration of heavier oligomeric silicate species in the activator solution, thereby significantly elevating its viscosity [41]. This increase in viscosity reduces the overall workability of the paste. There is no linear relationship between viscosity and flow, as measured via the diameter [42].

In a related study, Bondar [43] reported that the Na_2_O content (4%, 6%, and 8%) and silicate modulus of the activator solution (0.75, 1, 1.5, and 2) influenced the flowability of the alkali-activated slag. The water content was 0.47. At a given silicate modulus, increasing the Na_2_O dosage increased the slump of the alkali-activated slag (AAS) mixture. This effect was especially pronounced at a silicate modulus of 2.0, where an 8% Na_2_O dosage yielded a substantially greater slump than all other tested dosages and the corresponding OPC control.

### 3.4. Effect of Ms on Dynamic E-Modulus

**Figure 13:**
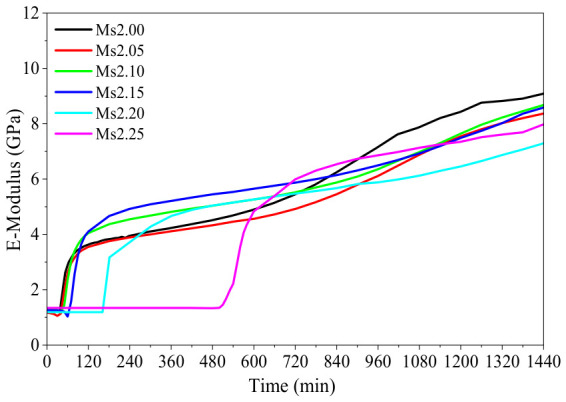
Evaluation of Young’s modulus of cylinder bar with activated-BFS paste at 20 °C.

The resonant frequency of the polycarbonate cylinder filled with activated BFS paste at room temperature was monitored for 1440 min after casting. The Young’s modulus of the activated-BFS paste in the cylinder was calculated based on the extracted values of the stiffness of the activated-BFS paste from the total stiffness of the cylinder, plus the activated-BFS paste, compared with that of the empty cylinder. Using this data, the curve of E-modulus versus curing days was constructed, as shown in Figure 13.

At the beginning of the reaction, the dynamic modulus curve remained almost flat because the fresh paste behaved like a viscous suspension rather than a solid. As network formation advanced, the curve rose sharply, indicating the rapid formation of the initial C-A-S-H network, which marks the transition from a fluid to a solid skeleton. After this rapid increase, the curve enters a plateau region, reflecting the stabilization of the early age structure. The samples setting first exhibited the lowest E-modulus in this plateau region. This behavior may be interpreted in light of the structural role of aluminum in C-A-S-H systems. The structural flexibility associated with multiple Al coordination states (Al-IV, Al-V, and Al-VI), as reported in [44] contributes to local structural variability within the network, reflecting the ability of aluminum to adopt dynamically changing coordination environments during early reaction stages. This coordination variability, together with the angular flexibility associated with Al–O bonding environments, is consistent with increased local structural heterogeneity at the nanoscale, and enhanced chain rearrangement and branching, potentially influencing the ordering and connectivity of the forming network.

In this context, early incorporation of aluminum and the associated structural variability may lead to a more disordered and dynamically evolving initial network, which could reasonably explain the comparatively low E-modulus observed in the early-age plateau region of the high-alkalinity mixes. Such effects are expected to be more pronounced in highly alkaline systems (Ms = 2.00–2.05), where rapid dissolution and early Al incorporation into the evolving C-A-S-H structure occur.

This interpretation therefore remains indirect. Establishing a direct correlation between Al coordination environments, nanoscale disorder, and macroscopic mechanical response would require dedicated, coupled microstructural and mechanical investigations capable of resolving both Al coordination states and the evolution of the nanostructure across relevant length scales.

A second, slower increase in stiffness was observed at later ages, which became sharper with decreasing Ms (increasing alkalinity). One possible explanation for this behavior could be related to the continued dissolution of slag particles and the progressive reaction of previously unreacted slag, leading to further network formation and densification of the matrix [26]. For Ms 2.25, this cannot be observed in the graph, but after 28 days, all samples obtained an E-modulus of approximately 15 GPa. The normalization of the curves versus time (for instance, the setting time) does not render the same profile (see Supplementary Information Figures S1–S3). This contrasts with the findings of Naqi [45], who reported that upon time normalization, the evolution of the ultrasonic P-wave velocity collapses onto a unique master curve, suggesting similar hydration and structuration kinetics regardless of the mixture composition.

### 3.5. Comparison Between Vicat Setting Times, Ultrasonic Measurements and IET and Reactivity

**Figure 14:**
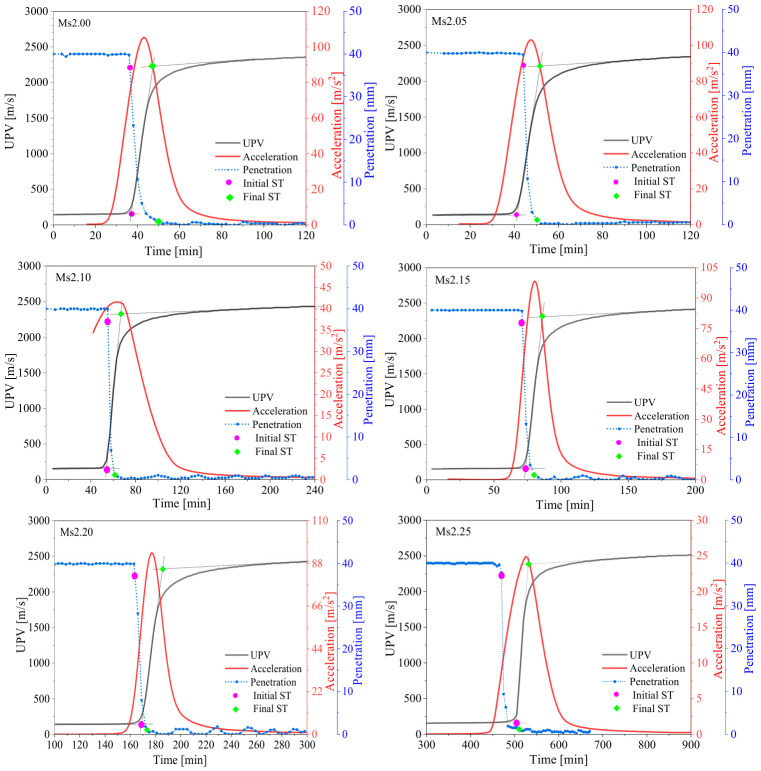
The ultrasonic velocity profile, its derivative (acceleration), and Vicat penetration depth curves for mixtures up to Ms 2.25.

**Figure 15:**
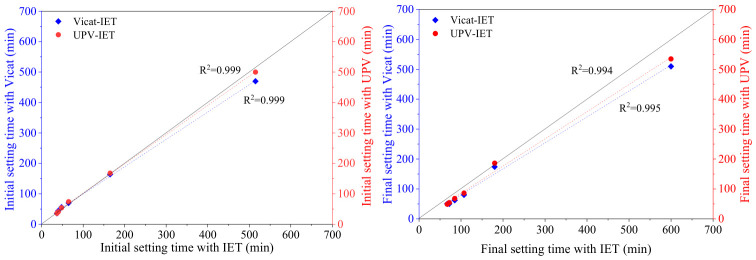
Correlation of setting time between different techniques (Vicat, UPV, and IET). Left: initial setting time, Right: final setting time.

The ultrasonic velocity profiles, their derivatives (accelerations), and Vicat penetration depth curves for all mixtures with moduli of 2.00 to 2.25 are presented in Figure 14. The initial and final setting points measured using the ultrasound and Vicat penetration methods are also shown in this figure. For all mixtures, there was a good correlation between the final setting time measured using the Vicat apparatus and the maximum acceleration point (UPV) for all pastes (Figure 15). Table 5 provides a detailed summary of the initial and final setting times for GGBFS-based alkali-activated pastes with Ms values ranging from 2.00 to 2.25, measured using Vicat, UPV, the maximum acceleration value, and IET.

The evolution of setting was evaluated using three different measurement techniques—Vicat, UPV, and IET—and the results were statistically compared through one-way ANOVA for both the initial and final setting times. For the initial setting time, the three methods produced very similar mean values, and the ANOVA confirmed that the small differences observed were not statistically significant (*p* ≈ 0.998). This indicates that all three techniques capture the onset of setting consistently, and the scatter within each method is much larger than any systematic difference between them. A similar trend was observed for the final setting time. Although the mean values increased as expected with the progression of hydration, the variability within each method again dominated the dataset, leading to a non-significant ANOVA result (*p* ≈ 0.959). Taken together, these findings show that Vicat, UPV, and IET provide mutually consistent assessments of both the beginning and completion of setting. The agreement across methods supports the use of UPV and IET as reliable, non-destructive alternatives to the traditional Vicat test for monitoring the development of stiffness in alkali-activated slag systems (Further information, including detailed tables (Tables S1–S4), is provided in the Supplementary Information to this article).

**Figure 16:**
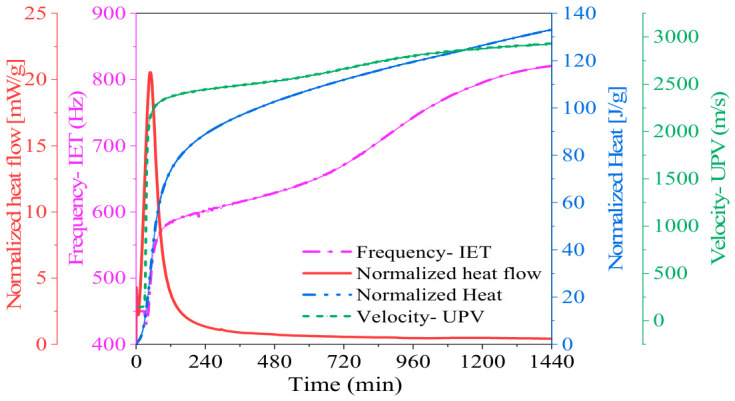
Comparison of isothermal calorimetry, UPV, and IET for activated paste with Ms 2.

The combined calorimetry, UPV, and IET results in Figure 16 illustrate how the chemical and mechanical processes evolved differently during the early reaction of the alkali-activated slag. The heat flow curve exhibited a typical sharp exothermic peak followed by a rapid decline. As the heat flow approached zero, the cumulative heat began to level off, suggesting that most of the exothermic reactions had already occurred.

In contrast, both the UPV and IET continued to rise long after the calorimetric signal became negligible. The second step in frequency, and thus E-modulus, increase between 700 and 1200 min is also visible with UPV, but not in calorimetry. This behavior reflects the fact that mechanical properties do not depend solely on ongoing heat-releasing reactions but also on microstructural processes with little or no heat effects. The close agreement between the UPV and IET results confirms that both techniques capture the same stiffness development, whereas calorimetry captures only the major chemical processes. Together, these results show that the paste continues to form a stronger and more connected solid network even after the main exothermic reactions are complete.

### 3.6. Effect of Ms on Compressive and Flexural Strength

**Figure 17:**
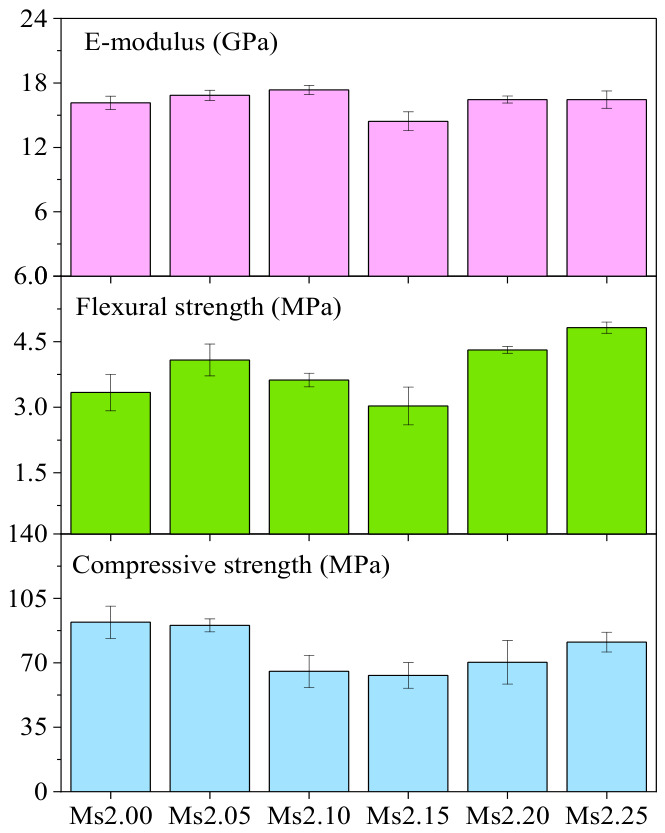
The effect of alkali activator modulus on mechanical properties at 28 days. E-modulus measured with IET.

The results shown in Figure 17 indicate a nonlinear relationship between the silicate modulus and the mechanical performance at 28 days. The compressive strength of cementitious pastes with silicate moduli of 2 and 2.05 was slightly higher than that of other pastes, but compositions with silicate moduli ranging from 2.15 to 2.25 exhibited minimal variation, with values converging around 65 MPa at 28 days. Flexural strength exhibited a more pronounced fluctuation across the modulus range, but it was low, even less than 10% of the compressive strength, which may reflect microcracking or surface defects in individual specimens. Aydin and his colleague [46] reported that in alkali-activated slag (AAS) mortars activated with a combination of sodium silicate and sodium hydroxide, increasing the silicate modulus (Ms) did not lead to an improvement in flexural strength. This behavior is attributed to the increased formation of microcracks within the matrix as the Ms value increases. In contrast, the elastic modulus, which was measured by IET, remained essentially constant at around 15 GPa, indicating that stiffness development is less sensitive to changes in defects or activator composition. The results indicate that the mechanical behavior of the system reflects the combined influence of several interacting processes rather than a single controlling factor. It also shows that producing decent samples with pure matrix is not evident and that the addition of fillers to mediate crack formation is essential. The nearly constant E-modulus (~15 GPa) across different silicate moduli suggests that the stiffness development of potassium-activated slag paste is governed primarily by the formation of a continuous reaction product network rather than by minor variations in activator composition or in microdefects. This indicates that once a continuous solid reaction network is established, further changes in chemistry mainly affect strength rather than elastic response.

## 4. Conclusions

In this study, the influence of the silicate modulus of the potassium silicate activator on key fresh and hardened properties was systematically evaluated, including reactivity, setting time, flowability, and mechanical performance (E-modulus, compressive strength, and flexural strength). The results demonstrated that increasing the activator modulus while maintaining a constant water content led to a clear retardation effect on the reaction kinetics, as evidenced by the delayed reactivity and prolonged setting times. This behavior is explained by the reduced availability of OH^−^, approximately a factor of 10 in the study, the most important reactive species for the breakdown of the BFS network, and consequently, this leads to slower structural build-up at higher modulus values.

Based on the calorimetry results, the initial and final setting times occurred after the reaction started and finished before it reached the maximum peak. Therefore, a strong relationship was observed between the setting times determined by the Vicat needle test, IET, and ultrasonic measurements and the corresponding reactivity curve. The initial and final setting times were closely aligned with the length of the dormant phase and the point at which the maximum acceleration was reached. It is also remarkable that the setting occurs rather quickly after the initial setting, even though the dormant period is long.

The flowability was also significantly affected, showing a consistent decrease with increasing modulus. Rheometer measurements supported these findings, confirming that higher-modulus mixtures exhibited increased viscosity and reduced workability. This indicates that the modulus plays a critical role in governing the rheological behavior of the system, directly impacting its processability in practical applications.

In contrast, the 28-day mechanical performance did not exhibit a consistent or systematic dependence on silicate modulus. Within the investigated range, this suggests that silicate modulus does not show a clear or dominant controlling influence on stiffness and strength development in potassium-activated slag pastes once a continuous load-bearing reaction product network has formed.

This behavior is consistent with previous studies on alkali-activated slag systems, which used combinations of sodium silicate and sodium hydroxide as activators, where hardened properties are governed by a combination of reaction degree, pore structure, and microstructural heterogeneity rather than a single compositional parameter [47,48,49].

The observed scatter in compressive strength is attributed to intrinsic variability in paste systems, where local differences in porosity and defect distribution can significantly influence mechanical response. Flexural strength was comparatively low and scattered, which is consistent with the high sensitivity of paste-only specimens to surface defects, entrapped air voids, and microcracking [47]. However, no direct microstructural or imaging evidence (e.g., SEM or optical microscopy) is available in this study to explicitly confirm these features; therefore, this interpretation remains indirect.

The results further highlight the potential role of fillers or aggregates in mitigating defect formation and improving resistance to microcracking during setting and drying [47]. This aspect will be addressed in future work. In addition, the absence of a clear long-term dependence on silicate modulus contrasts with its stronger influence on fresh-state properties such as workability and setting behavior, indicating that different governing mechanisms dominate fresh and hardened states. While silicate modulus influences early-stage solution chemistry and reaction kinetics, the hardened mechanical response appears to be more strongly controlled by the resulting microstructure, including porosity and defect distribution.

Overall, the findings highlight that the modulus is a key parameter in controlling early age behavior, particularly reactivity, setting characteristics, and flowability, whereas its impact on mechanical performance is less significant. The results also emphasize that a more detailed microstructural investigation is required to fully resolve the relationship between mix design, defect formation, and mechanical properties in potassium-activated slag pastes. These insights provide valuable guidance for optimizing the mixture design, where a balance between workability, setting behaviors, and performance requirements must be carefully considered based on the intended application.

## Figures and Tables

**Table 1:** Chemical composition of GGBFS obtained with XRF.

Content	CaO	SiO_2_	Al_2_O_3_	MgO	SO_3_	TiO_2_	K_2_O	Fe_2_O_3_	Na_2_O	MnO
**GGBFS (wt. %)**	40.9	34.6	12.3	8.1	1.4	1.0	0.5	0.5	0.4	0.2

**Table 2:** The physical properties of the GGBFS.

Type	Relative Density (g/cm^3^)	Blaine (cm^2^/g)	Particle Size Distribution (µm)			
			Mean	d_10_	d_50_	d_90_
**GGBFS**	2.9	4500	13.5	1.0	9.9	31.8

**Table 3:** Mixture design of alkali-activated GGBFS. The activator to GGBFS mass ratio is 0.8. Water to GGBFS mass ratio is 0.47. Ms is the ratio of SiO_2_/K_2_O.

Mix No.	GGBFS (%)	Ms (Solution)	K_total_/Al	Ca/Si_total_	K_total_/Si_total_	Al_total_/Si_total_
1	100	2.00	1.42	0.802	0.378	0.266
2	100	2.05	1.40	0.801	0.371	0.265
3	100	2.10	1.38	0.799	0.363	0.263
4	100	2.15	1.35	0.797	0.357	0.264
5	100	2.20	1.33	0.795	0.350	0.263
6	100	2.25	1.31	0.793	0.344	0.263
7	100	2.30	1.29	0.792	0.337	0.261
8	100	2.35	1.27	0.790	0.332	0.261

**Table 4:** pH of the silicate solution with different modulus.

Silicate Modulus	Ms2.00	Ms2.05	Ms2.10	Ms2.15	Ms2.20	Ms2.25	Ms2.30	Ms2.35
pH	13.79 ± 0.01	13.58 ± 0.04	13.34 ± 0.01	13.23 ± 0.01	13.12 ± 0.01	12.96 ± 0.03	12.84 ± 0.01	12.75 ± 0.03

**Table 5:** Setting time measurements of Vicat, UPV, and IET (initial and final setting time are selected based on the inflection points method, 50% of final points, and maximum acceleration). The reported value is the mean of three independent measurements, with a relative standard deviation (RSD) of 5–15%.

Mix ID	Initial Setting Time			Final Setting Time			
	Min			Min			
	Vicat	UPV	IET	Vicat	UPV	UPV (Max. Acce.)	IET
Ms2.00	36	36	37	47	49	44	67
Ms2.05	44	41	40	50	54	48	72
Ms2.10	56	54	48	62	68	63	85
Ms2.15	70	74	65	80	86	80	107
Ms2.20	164	168	165	174	186	177	180
Ms2.25	470	500	515	510	535	525	600

## Data Availability

The original contributions presented in this study are included in the article/Supplementary Materials. Further inquiries can be directed to the corresponding author.

## Supplementary Materials

The following supporting information can be downloaded at: https://www.mdpi.com/article/10.3390/ma19122604/s1, Figure S1: The E-modulus of different activated pastes vs normalized time; Figure S2: The Velocity of different activated pastes vs normalized time; Figure S3: The heat flow of different activated pastes vs normalized time. Table S1. Summary of statistical parameters for three experimental groups (initial setting time). Table S2. One-way ANOVA results comparing the initial setting times determined by different measurement methods. Table S3. Summary of statistical parameters for three experimental groups (final setting time). Table S4. One-way ANOVA results comparing the final setting times determined by different measurement methods.

## Abbreviations

The following abbreviations are used in this manuscript:

TRC	Textile-reinforced cement/concrete
AAS	Alkali-activated slag
OPC	Ordinary Portland Cement
GGBFS	Ground granulated blast furnace slag
NaOH	Sodium hydroxide
Na_2_CO_3_	Sodium carbonate
KOH	Potassium hydroxide
AAMs	Alkali-activated materials
Ms	Modulus
UHPC	Ultra-high-performance concrete
UPV	Ultrasound pulse velocity
IET	Impulse excitation technique
ST	Setting time
C-A-S-H	Calcium aluminosilicate hydrate
