# Supplementary material for: Optimizing Potassium-Based Activator Formulation for Balanced Reactivity, Flowability, Setting Time and Mechanical Performance of Alkali-Activated Materials
Source: Materials (Basel). 2026 Jun 17;19(12):2604. doi: 10.3390/ma19122604 (PMC13304399; doi:10.3390/ma19122604)
Supplement: Supplementary file 1 [file materials-19-02604-s001.zip › materials-4345902-supplementary.pdf]

Supplementary Materials: The following supporting information can be downloaded at: https://www.mdpi.com/article/doi/s1,s2,s3, Figure S1: The E-modulus of different activated pastes vs normalized time. Figure S2: The Velocity of different activated pastes vs normalized time. Figure S3: The heat flow of different activated pastes vs normalized time.

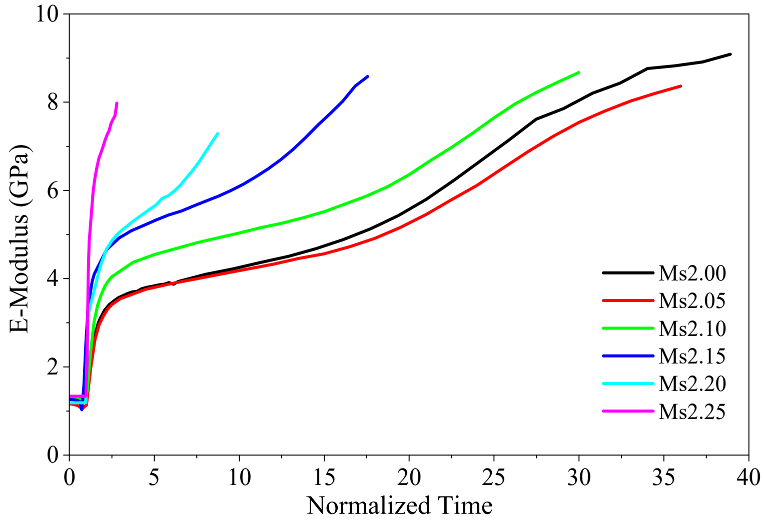

Figure S1. The E- modulus of alkali activated pastes (using IET) vs normalized time by initial setting time

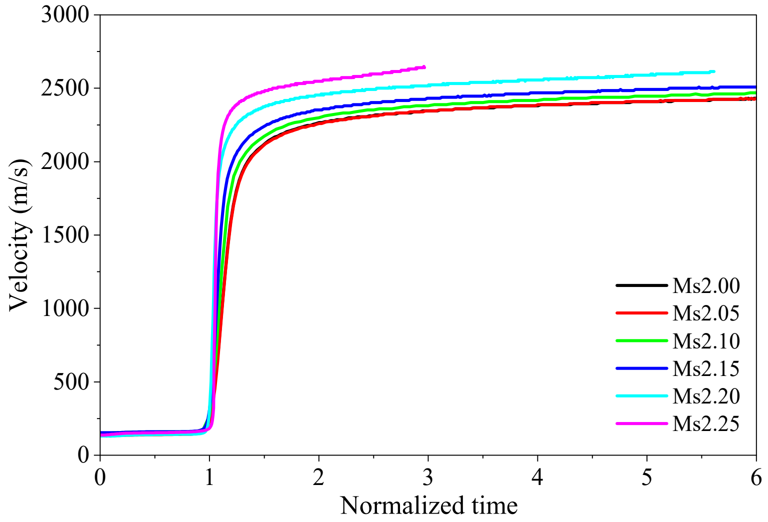

Figure S2. The velocity of alkali activated pastes (using UPV) vs normalized time by initial setting time

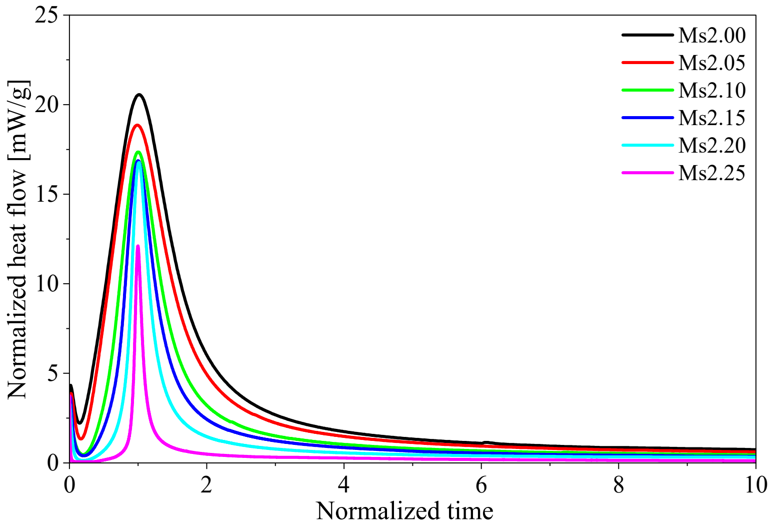

Figure S3. The heat flow of alkali activated pastes (using isothermal calorimetry) vs normalized time by initial setting time.

Table S1. Summary of statistical parameters for three experimental groups (initial setting time).

Groups	Count	Sum	Average	Variance
Column 1	6	840	140	28292.8
Column 2	6	873	145.5	32514.3
Column 3	6	870	145	35159.6

Table S2. One-way ANOVA results comparing the initial setting times determined by different measurement methods.

Source of Variation	SS	df	MS	F	P-value	F crit
Between Groups (methods)	111	2	55.5	0.001735	0.998267	3.68232

Table S3. Summary of statistical parameters for three experimental groups (final setting time).

Groups	Count	Sum	Average	Variance
Column 1 (Vicat)	6	923	153.8	32668.2
Column 2 (UPV)	6	978	163.0	35748.8
Column 3 (IET)	6	1111	185.2	43005.4

Table S4. One-way ANOVA results comparing the final setting times determined by different measurement methods.

Source of Variation	*SS*	*df*	*MS*	*F*	*P-value*	*F crit*
Between Groups (methods)	3114.3	2	1557.167	0.041926	0.959053	3.68232
